# The Involvement of the Proamnion in the Development of the Anterior Amnion Fold in the Chicken

**DOI:** 10.1371/journal.pone.0092672

**Published:** 2014-03-19

**Authors:** Ana de Melo Bernardo, Susana M. Chuva de Sousa Lopes

**Affiliations:** Department of Anatomy and Embryology, Leiden University Medical Center, Leiden, The Netherlands; New York Medical College, United States of America

## Abstract

The amnion was one of the most important evolutionary novelties in the animal kingdom, allowing independence of water for reproduction and subsequent exploration of terrestrial habitats, and is therefore an important structure to understand evolution. We have studied chicken amniogenesis using *ex ovo* culture systems and 3D-reconstructions of serially sectioned chicken embryos. We provide evidence for a transient depression of the head in the proamnion, forming a pouch, that positions the extraembryonic membranes dorsal to the head and that is fundamental for the correct formation of the amnion and chorion membranes. When this “sinking” process in the proamnion was blocked, the amnion/chorion did not form, even though the growth of the embryo per se seemed unaffected. Here, we give insight in the role of the proamnion in amniogenesis.

## Introduction

The appearance of the amniote egg, an evolutionary novelty from the amphibian ancestors of the reptiles, opened new evolutionary paths. The amniote egg contained all the materials needed for the embryo to develop: sufficient water, nutrients and energy. Only oxygen and heat were still required from the environment. Subsequently, animals became independent of water for reproduction and took the opportunity to explore terrestrial habitats. The original pattern of the amniote egg, that comprised the formation of four extraembryonic sacs (amnion, chorion, yolk sac and allantois), was such an important novelty that it remained conserved between reptiles, birds and mammals (reviewed in [1], [2]). In mammals, even in the absence of large amounts of yolk, the developing of the four extraembryonic “sacs” has been retained to a certain extent (reviewed in [1], [2]).

In chicken, the generation of the extraembryonic sacs takes place after gastrulation, with the appearance of the extraembryonic coelomic cavity in the extraembryonic mesoderm (ExM) [3]–[6]. This cavity contributes to the separation of two major extraembryonic tissue layers: the splanchnopleure formed by endoderm and ExM; and the somatopleure formed by ectoderm and ExM. The splanchnopleure develops into a complex system of blood vessels, the yolk sac, responsible for supplying yolk and egg white materials to the embryo (nourishment); and it will architect the allantois, a structure connected to the primitive gut, which stores toxic by-products produced by the embryo. On the other hand, the somatopleure gives rise to both the chorion and the amnion. The chorion will allow gas exchanges with the external environment, while the amnion constitutes a protective membrane that surrounds the embryo and prevents its desiccation.

Interestingly, the ExM does not populate the extraembryonic area immediately anterior to the chicken foregut (and the developing heart), the so-called proamnion, but as the ExM spreads anteriorly it does so by circumventing the proamnion with two separate lateral wings that fuse axially. The proamnion remains diblastic composed only of ectoderm and endoderm and during the presomitic stages (until Hamburger and Hamilton stage (HH)7 [7]) it has been shown to express retinoic acid receptor isoform β2 (RARβ2) [8]. The proamnion, as diblastic structure, disappears gradually [9], [10], however according to Rosenquist (1971), endoderm fate-mapped to the proamniotic region can become incorporated in the ventral foregut and midgut [11]. The proamnion should not be confused with the buccopharyngeal membrane, another cranial diblastic membrane, present in both human and chick embryos, that gives rise to the opening of the oral cavity [12], [13].

The current model of amnion development in chicken describes the separation between the amnion and the chorion from four distinct folds of somatopleure: the anterior amnion fold, two lateral amnion folds and the posterior amnion fold [7], [9], [14]–[16]. The growth of the anterior amnion fold would create sufficient tension to elevate the somatopleure, subsequently leading to the formation of the two lateral amnion folds [17]. The posterior amnion fold surrounds the caudal region, similarly to the anterior amnion fold, but growing in opposite direction with an 18 hour delay. The embryo becomes enclosed (by amnion and chorion), after the fusion of the four different amniotic folds over the dorsal side of the embryo by 72 hours of incubation [15].

Recently, we have investigated the migratory route of the primordial germ cells (PGCs) in chicken embryos from the germinal crescent region of the yolk sac to the genital ridges and noticed that the PGCs would often be situated on an extraembryonic membrane clearly positioned above (or dorsal to) the head of the embryo [18]. As classically the PGCs are localized in the splanchnopleure and later in the vasculature of the yolk sac [19], we were surprised by their dorsal localization and we have investigated the formation of both the anterior amnion fold and the yolk sac between HH10 and HH13 by culturing embryos *ex ovo* and by performing 3D-reconstructions of serially sectioned chicken embryos. We concluded that the developing head at HH13 is submerged in a pouch formed by the diblastic proamnion. This displacement of the head positioned both the splanchnopleure (yolk sac and hence the PGCs) and the somatopleure transiently in a dorsal position to the head, explaining our observations of PGCs present dorsally to the head [18]. In 1889, an anatomical study by Shore and Pickering suggested the importance of the proamnion in the formation of the anterior amnion fold [9], but their model has been ignored in most textbooks to date, where the proamnion is not depicted. We now experimentally show that the depression of the head in the proamniotic pouch is paramount for the correct position of the anterior amnion fold, as without this depression of the head in the proamnion, the somatopleure is unable to elevate sufficiently to cover the extending embryo and to surround the embryo generating both the amnion and the chorion.

## Results and Discussion

### The Head Sinks in Proamnion, Positioning both the Somatopleure and Splanchnopleure Dorsally

**Figure 1:**
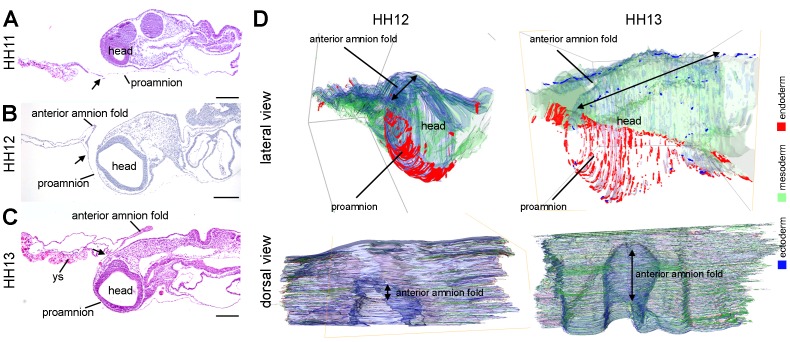
The head tip submerged progressively in proamnion between HH11–13. (A–C) Medial sagittal sections stained with H&E of chicken embryos at HH11 (A), HH12 (B) and HH13 (C). Black arrow points to the junction between proamnion, splanchnopleure/yolk sac and somatopleure/amnion. (D) Lateral and dorsal views of 3D reconstruction of serial transversal sections of embryos at HH12 and HH13 (sectioned plane) with the different germ layers in different colors (ectoderm in blue, mesoderm in green, endoderm in red). Double headed arrows indicate the developing anterior amnion fold. Abbreviations: ys, yolk sac. Scale bars: 200 μm.

To understand the formation and position of the anterior amnion fold and yolk sac in chicken embryos, we isolated chicken embryos at HH11, HH12 and HH13 containing the extraembryonic membranes and serially sectioned them sagittally and transversally. At HH11, the embryo head rested on top of the proamnion (Figure 1A) and as development proceeded, the anterior amnion fold is formed starting to involve the tip of the head and the head progressively submerged in the proamnion, forming a pouch, at HH12-HH13 (Figure 1B,C). The point of separation between proamnion (endoderm/ectoderm), splanchnopleure (endoderm/mesoderm) and somatopleure (ectoderm/mesoderm) was clearly visible at HH11, HH12 and HH13 (black arrow in Figure 1A–C). At HH13, the proamnion covered the forebrain and as a consequence both the splanchnopleure and somatopleure were elevated and positioned dorsally from the forebrain, facilitating the growth of the amniotic fold to cover the midbrain (Figure 1C).

**Figure 2:**
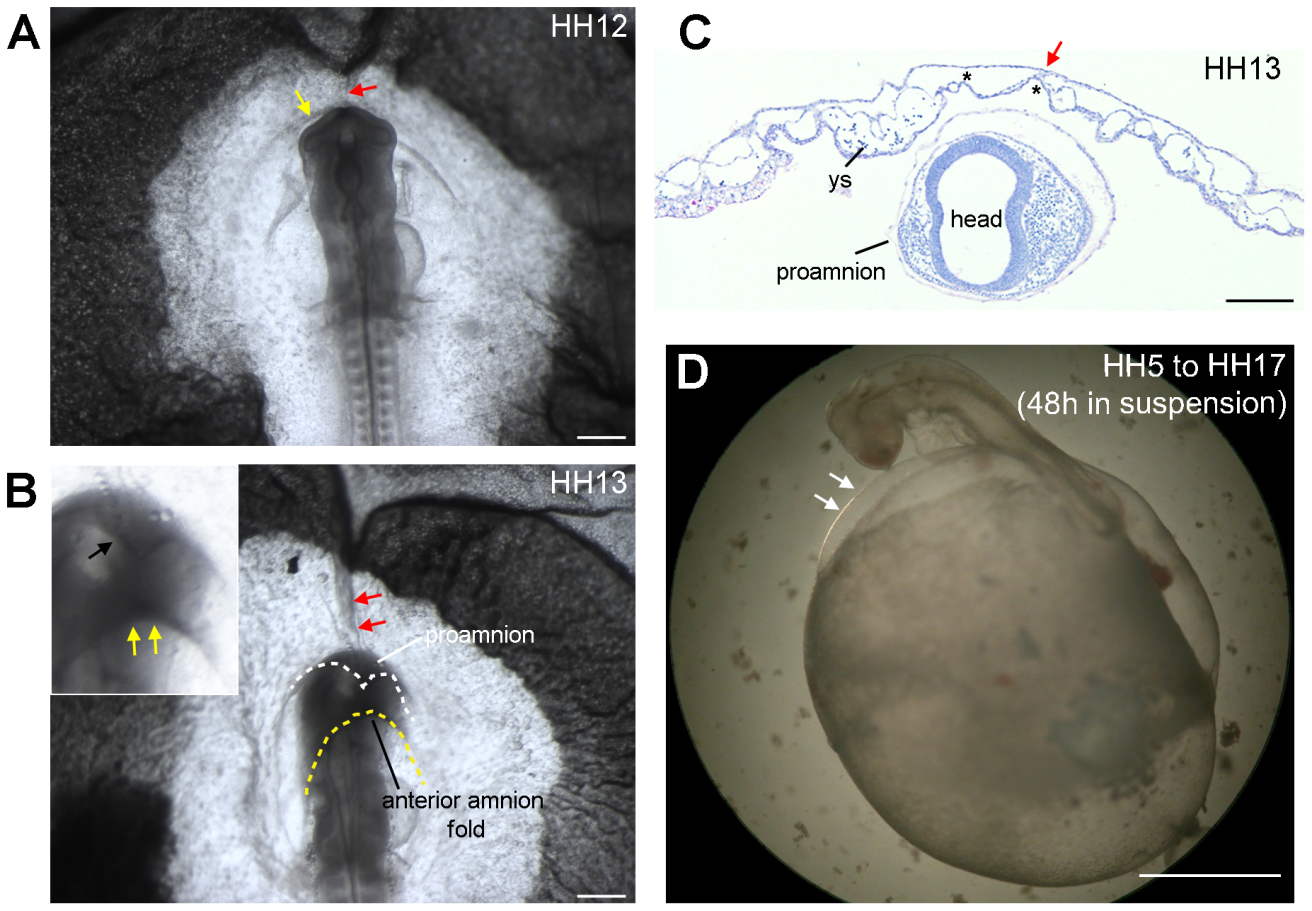
Passive displacement of somatopleure and splanchnopleure due to the sinking of the head in proamnion. (A) Whole mount HH12 chicken embryo with the anterior amnion fold forming (yellow arrow) and an axial strip visible from the proamnion until the area opaca (red arrow). (B) Whole amount HH13 chicken embryo showing the head sinking in the proamnion, which already covers the tip of the head (white dotted line and black arrow in the magnified view in the top left corner), and that the anterior amnion fold has developed posteriorly (yellow dotted line and yellow arrows in the magnified view in the top left corner). (C) Transversal section of a HH13 chicken embryo showing the head completely surrounded by proamnion and the avascular region of splanchnopleure/yolk sac (in between asterisks) attached to the somatopleure by a double layer of mesoderm (red arrow). (D) In embryos cultured *ex ovo* “in suspension”, from HH5 to HH17 (for 48 hours), the head did not sink in proamnion and therefore the formation of the amnion was impaired. White arrows point to the somatopleure. Abbreviations: ys, yolk sac. Scale bars: 500 μm (A, B), 200 μm (C) and 1 mm (E).

In the absence of known molecular markers for the proamnion at HH12-HH13, we performed 3D reconstructions of serial transverse-sectioned embryos to visualize the boundaries of the proamnion in more lateral positions to the head (Figure 1D). The reconstructions showed that the proamniotic domain extended laterally between HH12 and HH13 as the head submerged (Figure 1D). In addition, the dorsal view of the 3D reconstructions showed that the anterior amnion fold is growing dorsally and progressively in an anteroposterior direction between the two stages (Figure 1D, dorsal view), while the head is sinking in proamnion (Figure 1D, lateral view). A detailed analysis of whole mount embryos of HH12-HH13 (Figure 2A–B) also revealed that the head indeed submerged in a proamniotic pouch, positioning the boundary between the proamnion, the splanchnopleure/yolk sac and the developing anterior amnion fold dorsally from the head (Figure 2A and white dotted line in Figure 2B), allowing the dorsal expansion of the anterior amnion fold in a posterior direction (yellow arrows in Figure 2B). Histological analysis of HH13 embryos sectioned transversally revealed that the tip of the head was indeed completely surrounded by proamnion (Figure 2C).

In 1951, Hamburger and Hamilton described the presence of the anterior amnion fold covering the forebrain’s region at HH12 and extending to the midbrain and the anterior part of the hindbrain at HH13 [7], indicating a directional growth from anterior to posterior. The proamnion was not mentioned in that classical paper.

### The Splanchnopleure/Yolk Sac Contains an Avascular Strip above the Head at HH13

We have noticed an undescribed feature in the area pellucida at HH12-HH13: a prominent axial strip of splanchnopleure/yolk sac (red arrow in Figure 2A) as the embryo submerged under the anterior amnion fold. In HH13 whole mount embryos, this axial strip enlarged, extending from the border of the proamnion until the area opaca (red arrows in Figure 2B). Moreover, the axial strip of splanchnopleure/yolk sac that was present above the head was free of developing vasculature in contrast to the more lateral splanchnopleure/yolk sac (between asterisks in Figure 2C) and that the avascular region of the splanchnopleure/yolk sac remained consistently attached to the somatopleure by a thin double layer of mesodermal cells, known as the median mesoblastic septum (red arrow in Figure 2C). This septum, also observed in reptiles, is the consequence of the growth of the two lateral mesodermal wings axially in front of the proamnion and the formation of the right and left extraembryonic coelom, keeping them physically separated axially [10], [20].

### When Cultured “in suspension” the Amnion/Chorion did not Develop Properly

In 1948, in a series of experimental procedures on the development of the amnion in chicken embryos, Adamstone and colleagues showed that the cauterization (burning using a hot needle) of a limited group of cells in the proamnion, prevents the development of the anterior amnion fold [21], suggesting the involvement of the proamnion in this process.

To understand whether the depression of the head in the proamnion was necessary for the development of the anterior amnion fold forming the amnion and chorion in the process, we cultured chicken embryo using a modified Cornish pasty method [18], [22], a “suspension” culture system, whereby the embryos were cultured on top of a sealed mini yolk. In this method, the embryos were collected at HH5 and cultured until HH17 (for 48 hours) in suspension. Here, the head failed to submerge in the proamnion and as a consequence the somatopleure was not positioned dorsally to the head and therefore the amnion and chorion were unable to form properly (Figure 2D).

### Amniogenesis Occurred in Chicken Embryos Growing in an “non inverted” Position

An elegant *ex ovo* culture system, originally developed by Denis New and named after him, has been adapted and used in many studies on the development of chicken embryos [23], [24]. In this system, chicken embryos are grown with their ventral side facing upwards up to 48 hours and did not form the amnion. In 1963, Nicolet and Gallera made some modifications to the “New” culture system, using two glass rings instead of only one and an agar-based substrate, and compared the development of the blastoderm for 48 hours between an “inverted” and “non inverted” position [25]. Interestingly, when the blastoderm is grown in a “non inverted” position, the amnion developed and covered the embryo properly. Even though the formation of a “capuchon céphalique” (head pouch) in the “non inverted” grown embryos was mentioned, Nicolet and Gallera did not investigate further the anatomical nature of this structure [25].

**Figure 3:**
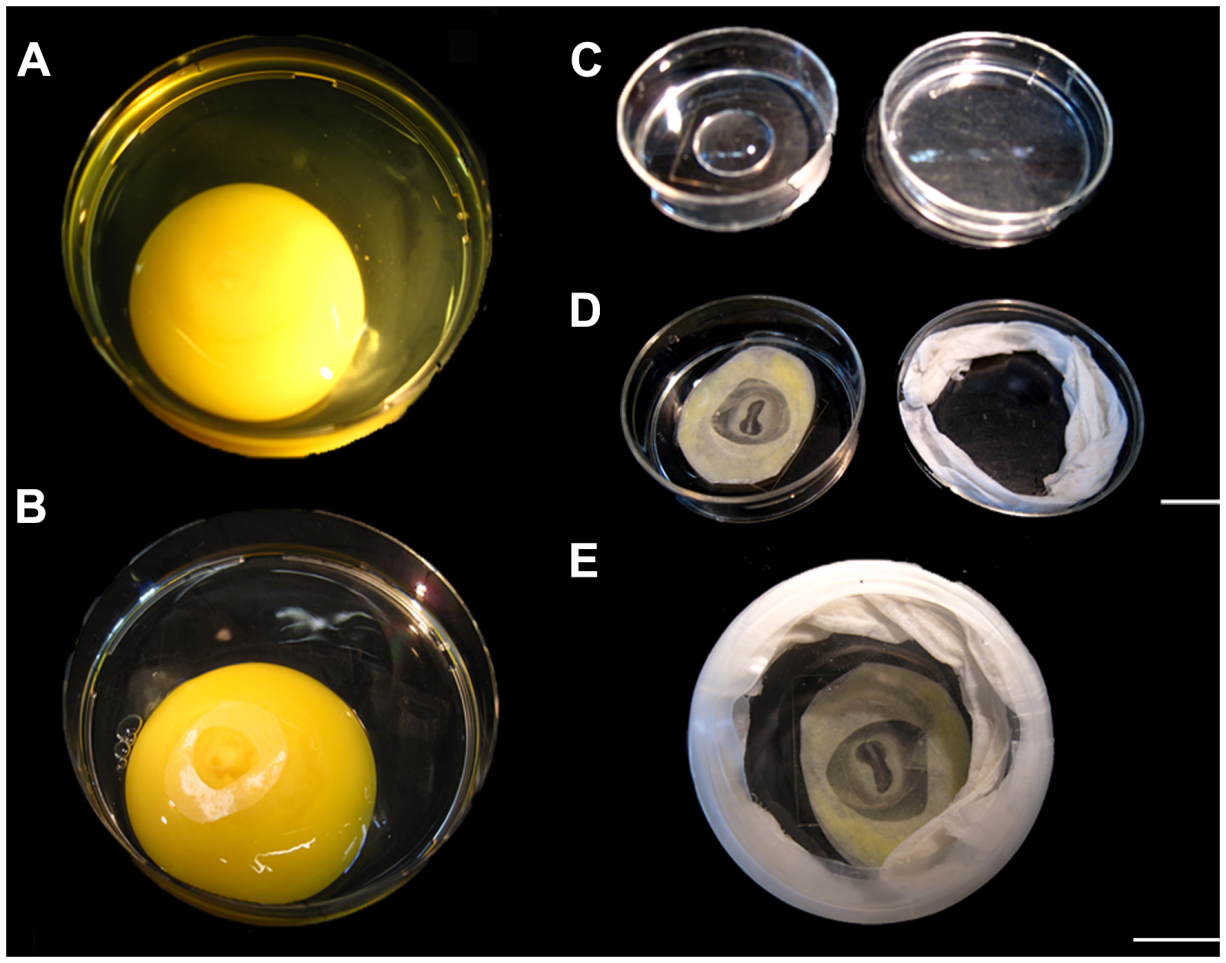
“Inverted and non inverted” *ex ovo* culture of chicken embryos. (A) The content of the egg was deposited into a petri dish, the thick albumen was removed and and the blastoderm was positioned upwards. (B) A piece of filter paper, with a central hole, was placed on the yolk, positioning the blastoderm in the central hole and the border of the filter paper with the embryo attached cut with scissors. (C) Before (B), a 500 μl drop of PBS was placed in a petri dish with a glass bottom. (D) The embryo attached to the filter paper was placed on the drop of PBS in an “inverted” or “non inverted” position. The lid of the petri dish was coated with (PBS) humidified paper before closing the petri dish. (E) The petri dish containing the embryo was sealed with parafilm. Scale bars: 1 cm in A,B,E and 1 cm in C,D.

**Figure 4:**
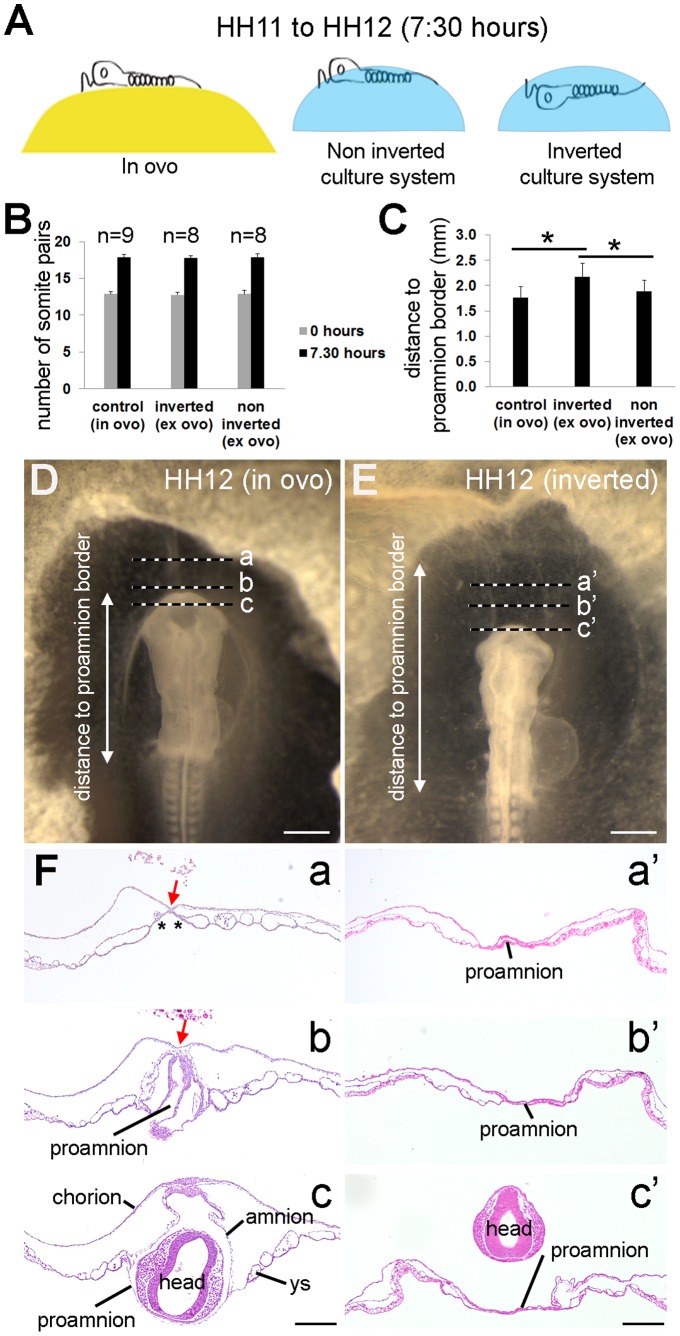
The anterior amnion fold did not develop in embryos growing “inverted”. (A) Scheme of the “inverted” culture system where HH11 chicken embryos were cultured for 7∶30 hours and compared with embryos growing *in ovo*. (B) Number of somite pairs at the start (0 hours) and after 7∶30 hours of incubation in each group. (C) Distance between the tip of the anterior intestinal portal and the border between the proamnion and the yolk sac in each group. *, *P*<0.05. (D-E) HH12 embryos growing *in ovo* (D) and in the “inverted” culture system (E) transversally sectioned (F) at the indicated levels (a–c and a’–c’). In the control embryo note the avascular region of splanchnopleure/yolk sac (in between asterisks in a) attached to the somatopleure by a double layer of mesoderm (red arrow in a and b). Abbreviations: ys, yolk sac. Scale bars: 500 μm (D,E) and 200 μm (F).

We hypothesized that the formation of the “capuchon céphalique” described by Nicolet and Gallera was in fact a consequence of the sinking of the head in proamnion. To test this we have developed a simple *ex ovo* culture system combining the use of filter paper (instead of glass rings) previously introduced by Chapman and colleagues [26] and using PBS solution as substrate for culture (instead of agar) (Figure 3, Figure 4A). We cultured HH11 chicken embryos containing the intact extraembryonic membranes with their ventral side facing either upwards (“inverted”) or downwards (“non inverted”), and compared those to embryos grown *in ovo* (Figure 3, Figure 4A). All embryos were allowed to grow for 7∶30 hours (from stage HH11 to stage HH12), the timing when the head submerges in proamnion *in ovo* (Figure 1A,B).

The number of somites was counted in each embryo in each condition, before and after the experimental procedure. Embryos growing “inverted” (n = 8) and “non inverted” (n = 8) *ex ovo* and *in ovo* control embryos (n = 9) formed similar number of somites during the experiment (5 pairs of somites) (Figure 4B). During chicken development, a new pair of somites emerges each 1∶30 hours [27] and therefore the expected number of new somite pairs formed in 7∶30 hours is 5, which was observed in all conditions. The formation of the expected number of somites provided evidence that the *ex ovo* culture system used (“inverted” and “non inverted”) did not interfere with the correct development of the embryo and therefore the abnormalities in amniogenesis observed in embryos grown “inverted” were perhaps caused by the positioning of the (normal) embryo head in relation to the proamnion.

### The Depression of the Head in Proamnion is Necessary for the Development of the Anterior Amnion Fold

To quantify differences in proamniotic extension in the embryos grown “inverted” and “non inverted”, we measured the distance between the tip of the anterior intestinal portal and the border between the proamnion and the yolk sac in embryos incubated in the different conditions (Figure 4C–E). Whereas both *in ovo* and “non inverted” cultured embryos showed a comparable proamnion (distance 1.77±0.21 mm and 1.86±0.22 mm respectively, P = 1); the “inverted” cultured embryos showed an extended proamnion (distance 2.18±0.27 mm), significantly different from the two other groups (Figure 4C), with a P = 0.005 to the control group and a P = 0,043 to the “non inverted” group.

Transversal sections of control embryos showed that the splanchnopleure/yolk sac remained attached to the somatopleure by a thin double layer of mesodermal cells (septum) (red arrow in Figure 4Fa–b) and those embryos also showed the avascular axial strip in the splanchnopleure/yolk sac (asterisk in Figure 4Fa). In the inverted group, the proamnion extended anteriorly, instead of folding into a pouch as in the control and “non inverted” group (Figure 4Fa’–c’ and data not shown). In the “inverted” group, both mesodermal wings formed a coelom, but they did not zipped anteriorly and, as a consequence, the anterior amnion fold was unable to form and the lateral amnion folds were underdeveloped and unable to surround the head to form either amnion or chorion (Figure 4Fa’–c’). In the control and “non inverted” group, the anterior amnion fold was visible and correctly positioned to give rise to the amnion and chorion (Figure 4Fc and data not shown). The downward movement of the head in these embryos into the proamnion is likely due to a combination of factors, including: tissue density, tissue folding induced by differential growth or tension distribution, and the specification of an ectodermal hinge point. Our anatomical analysis showed that indeed the proamnion plays a role in the development of the anterior amnion fold in chicken.

### Amniogenesis in Mammals

In mammals, the proamnion has been described in the rabbit [28], some species of bat [28] and in some species of marsupial [1]. It may be that depending on the type of blastocyst (existence of only mural or mural/polar trophectoderm) [29] and mode of implantation (and type of placenta) [30]–[32], the proamnion will be formed or not.

In mice, the amniochorionic fold is formed from the most posterior part of the primitive streak and grows (by cavitation) to fuse with the anterior part of the embryo during gastrulation [33]. There seems to be a transient diblastic region just anterior to the neuroectoderm at 7.5 days post coitum. This diblastic region may play a role as the developing head undergoes a ventral flexure repositioning the amniotic junction. However, this ventral flexure occurs after the amnion and chorion are formed.

In humans, having a similar type of blastocyst to the mouse that contains both polar and mural trophectoderm, there is no proamnion formation. In fact, early during human implantation and before gastrulation is initiated (or any mesoderm has been formed), the epiblast cavitates giving rise to amniotic cavity. The amnion will face the polar trophectoderm, while the epiblast will face the hypoblast. The human embryo completes the formation of the ectoderm part of the amnion by 9 days after conception. Later during gastrulation, the only diblastic structure is the buccopharyngeal membrane formed anteriorly (and the cloacal membrane posteriorly) [34].

### The Proamnion, an Evo-devo Perspective

**Figure 5:**
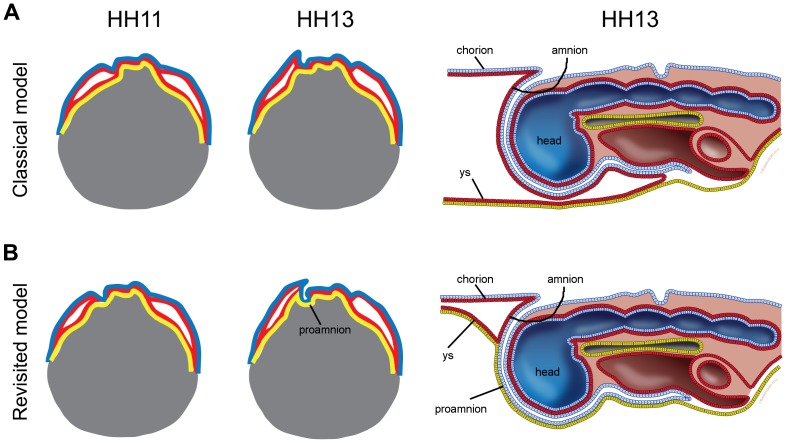
Two models (classical and revisited) for anterior amnion fold development in chicken embryos. (A) Sagittal scheme, representing the classical model, of HH11 and HH13 chicken embryos adapted from a Developmental Biology textbook [36] and a detail view of the head region at HH13. (B) Sagittal scheme, representing the revisited model, of HH11 and HH13 chicken embryos adapted from Shore and Pickering [9]. In both schemes, ectoderm is blue, mesoderm is red, endoderm is yellow and yolk is gray. Abbreviations: ys, yolk sac.

In the late 1800s, it was noted that different amniote species, including different species of birds [5], [9], [10], [35], reptiles [20], [35] and mammals [28], [35], share the initial steps of amniogenesis, regarding the initial sinking of the head in proamnion, positioning the somatopleure and splanchnopleure transiently above the head. In the late 1800s, some of those authors mention that “notable features are being overlooked” [20] and that “erroneous notions prevail at present day” as “classic series of diagrams constructed on this supposition being copied extensively by writers on embryology” [10] and is still the case today. We conclude that the proamniotic pouch is often being confused with the anterior amnion fold and that the position of the yolk sac, as commonly depicted in textbooks, is misplaced (Figure 5A,B).

## Methods

### Embryo Collection and Histology and 3D Reconstruction

Fertilized White Leghorn chicken (*Gallus gallus*) eggs were incubated in a humidified atmosphere at 37.0°C until HH11–13 [7]. The embryos were isolated (HH11 n = 6; HH12 n = 10; HH13 n = 13), fixed overnight in 4% paraformaldehyde, included in paraffin, sectioned and stained for hematoxylin and eosin (H&E) as previously described [18]. Sections were imaged on an Olympus AX70 microscope (Olympus) equipped with an Olympus XC50 camera (Olympus). For 3D reconstructions, serial paraffin transversal sections of freshly isolated embryos at stage HH12 and HH13 stained with H&E were digitalized using a Panoramic MIDI scanner (3D Histech) and reconstructed with Amira 4.1 software (Visage Imaging).

### *Ex ovo* Culture Systems

The “inverted” culture system was developed combining the “filter paper” method of Chapman and colleagues [26] with that of Nicolet and Gallera [25]. In our method, embryos of HH11 were isolated using a piece of filter paper with a central hole (+/−1 cm in diameter) placed onto the albumen with the embryo in the middle (Figure 3A–B). The border of the filter paper with the embryo attached was cut with small scissors and gently drawn away with forceps and pulled from the yolk in an oblique direction. The excess of yolk was then washed with PBS, while keeping the embryo attached to the filter paper. Thereafter, the embryos were placed with either their ventral (n = 14) or dorsal (n = 8) side facing upwards in a petri dish (MatTek) with glass bottom on a 500 μl drop of PBS (Figure 3C–D). The borders of the lid of the petri dish were first sealed with humid paper (with PBS) and then wrapped with parafilm to avoid evaporation (Figure 3D–E). Thereafter, the embryos (“inverted” group and “non inverted” group) were cultured for 7∶30 hours (until HH12) at 37°C with humidity on air. As control, an opening was made in the shell of control eggs with embryos at HH11 (n = 15), part of the vitelline membrane was removed to expose the embryo and some drops of PBS were added to avoid embryo drought. The eggs were then re-sealed with tape and incubated for 7∶30 hours at 37°C with humidity on air, and the embryos were collected afterwards. Somites were counted at the onset and the end of the culture period. The resulting embryos were isolated in PBS and some embryos were processed as described above for histology.

For the “suspension” culture system, embryos of HH5 (n = 10) were prepared for *ex ovo* culture essentially as described [18], [22]. Briefly, chicken embryos were grown on top of a mini yolk sac structure in a fish embryo-like shape. After 72 hours of incubation the embryos were analysed.

### Image Acquisition of Whole Mount Embryos and Statistical Analysis

A Leica M420 stereoscope (Leica, Rijswijk, the Netherlands) equipped with a Nikon E4500 Coolpix camera (Nikon, Tokyo, Japan) was used to image whole mount embryos. ImageJ was used to measure the distance between the tip of the anterior intestinal portal and the border between the proamnion and the yolk sac in the embryos cultured *ex ovo* “inverted” and “non inverted” and control embryos grown *in ovo*. IBM SPSS Statistics 20 was used to perform a one-way ANOVA test to compare the average of the distance in each of the three conditions. P<0.05 (*) were considered statistically significant.

## Conclusions

Here, we have investigated the role of the proamnion in the development of the anterior amnion fold leading to a correct amniogenesis in chicken embryos. We suggest using two different culture systems (“suspension” and “inverted/non inverted”) that when the chicken (tip of the) head is not able to sink in the proamnion, the anterior amnion fold is not placed properly above the head, impairing correct amniogenesis.
